# A Cross-Sectional Epidemiological Survey of Work-Related Musculoskeletal Disorders and Analysis of Its Influencing Factors among Coal Mine Workers in Xinjiang

**DOI:** 10.1155/2020/3164056

**Published:** 2020-08-08

**Authors:** Xianting Yong, Fuye Li, Hua Ge, Xuemei Sun, Xiaofan Ma, Jiwen Liu

**Affiliations:** Department of Occupational Health and Environmental Health, College of Public Health, Xinjiang Medical University, Urumqi, China

## Abstract

This study is to investigate the prevalence of work-related musculoskeletal disorders (MSDs) and the influencing factors among coal mine workers employed in on-site operations. The job burnout scale and MSD scale were implemented to investigate a random sample of 1,500 coal mine workers working in on-site operations in Xinjiang, China. In total, 1,325 valid questionnaires were collected, with a recovery rate of 88.33% (1,325/1,500). The rate of job burnout was 90%, of which 39.8% were categorized as mild burnout, 43.8% as moderate burnout, and 6.4% as severe burnout; the average job burnout score was 50.77 ± 11.93. The annual prevalence of MSDs was 65.6%, with the highest annual prevalence in the waist (50.7%), followed by the neck, shoulder, and knee, and the lowest prevalence in the elbow (18.8%). Of the areas of the body affected by work-related MSDs, the highest proportion of requests for leave of absence was related to the waist, accounting for 25.7% of requests, while the lowest proportion (13.4%) was related to the wrist. In addition, the incidence of MSDs increased with the years of service. The lowest incidence of MSDs was associated with the two-shift and three-group working pattern. The prevalence of MSDs in the neck and waist was higher in women than in men. The prevalence of MSDs in various body parts increased with the years of service. Moreover, multiple logistic regression indicated that three shifts with four groups (OR = 1.096, 95% CI: 0.832-1.445), working more than 10 years (OR = 3.396, 95% CI: 2.369-5.748), working more than 20 years (OR = 3.008, 95% CI: 1.419-6.337), significant bending (OR = 2.062, 95% CI: 1.400-3.038), forward neck tilting (OR = 1.572, 95% CI: 1.071-2.281), maximum force operation within a short period of time (OR = 1.7222, 95% CI: 1.164-2.547), repeated movement of upper arms or fingers (OR = 1.495, 95% CI: 1.034-2.161), slip or fall incidents (OR = 1.124, 95% CI: 1.039-1.216), work under conditions of cold or temperature variations (OR = 1.911, 95% CI: 1.342-2.720), mild burnout (OR = 1.492, 95% CI: 1.016-2.191), moderate burnout (OR = 1.852, 95% CI: 1.267-2.708), and severe burnout (OR = 2.001, 95% CI: 1.145-3.496) were risk factors for MSDs. In conclusion, there is a high annual prevalence of MSDs among the coal mine workers employed in on-site operations in Xinjiang, China. Measures to reduce this prevalence are required.

## 1. Introduction

Work-related musculoskeletal disorders (MSDs) refer to an injury or dysfunction of muscles, bones, nerves, tendons, ligaments, joints, cartilage, and spine [1]. It has been reported that 30% of European workers suffer from back pain, and the economic loss caused by back pain in Holland accounts for 1.7% of the national wealth every year. Furthermore [2, 3], the number of days absent from work due to MSDs accounts for 15%-22% of the total as a consequence of industry-related diseases in the Federal Republic of Germany every year. The direct and indirect economic losses incurred in Canada due to MSDs are as high as nearly 100 billion US dollars every year. Thus, the serious consequences of MSDs have gradually aroused widespread concern in economically developed countries [4]. Indeed, MSDs can also be induced by adverse psychosocial factors, which in turn have negative effects on psychological and social states and further increase the risk of workers, thus exhibiting a two-way connection and feedback loop between the two [5–7]. Under cognitive and emotional stress, a longer period of rest time is required to allow muscle relaxation [8]. Although coal mine workers are high-risk groups for MSDs [9–11], the influence of psychosocial factors in this group remains to be clarified. There is a wide range of psychosocial factors, including job demands, job control, job support, social support, and job satisfaction [12]. Furthermore, some studies have shown that, especially in developing countries, individual physical, social, and organizational risk factors are independent predictors of lumbar back symptoms (LBS), although on their interactions have seldom been explored. Previous studies have shown that psychosocial factors are more likely to lead to LBS than physical factors, among which the night shift and unreasonable shift systems are also risk factors [13, 14]. Additionally, a study in Indonesian coal miners showed interactions between lumbago symptoms and physical and psychological risk factors, with a higher incidence of lumbago symptoms among smokers and permanent night shift workers although the mechanisms remain to be clarified [15].

MSDs are a serious threat to health, working ability, and quality life and represent the primary reason for the decline in the working population aged from 20 to 55. In some industrialized countries, traditional occupational diseases caused by chemical toxicity, dust, and noise have been gradually controlled, while chronic MSDs of the waist, back, shoulder, neck, and wrist have become widespread occupational health problems associated with high economic losses [16]. Thus, the psychology and musculoskeletal health of coal mine workers in specialized environments require further investigation.

In China, the coal mine workers are in a large population alling into the category of specialized occupations as a consequence of the rich coal resources. Specifically, coal miners are engaged in long-term operations executed hundreds, or even thousands, of meters underground, where the working environment is complicated, with a high level of responsibility and long working hours, resulting in an unstable biological rhythm. In addition, the working environment contains occupational risk factors such as high temperature, noise, dust, vibration, and unreasonable working systems, which have adverse effects on the psychology of coal miners [17]. In this study, using a questionnaire survey, we investigated the prevalence of MSDs and related influencing factors among coal miners working in an on-site operation in Xinjiang. This information will provide a scientific reference for further improving the quality of life of coal miners and establishing a healthy and harmonious working environment.

## 2. Methods

### 2.1. Participants

This cross-sectional study was conducted via questionnaire using random cluster sampling. From March 2017 to December 2017, 1,500 coal mine workers employed in on-site operations at six coal mine enterprises in Xinjiang were randomly selected for inclusion in our study. The participants were aged 18 years or over and under 60 years with ≥1 year of service. A total of 1,500 questionnaires were distributed in this survey. After the exclusion of the invalid questionnaires, 1,325 valid questionnaires were finally returned, with a recovery rate of 88.33%.

### 2.2. Job Burnout

In this study, we used the job burnout scale revised by Li et al. [18] to measure job burnout. The questionnaire was designed to evaluate emotional exhaustion, depersonalization, and reduced sense of achievement, with five items included for each factor (15 items in total). The Cronbach *α*-coefficient of the questionnaire was 0.780 and the test-retest reliability was 0.843 [19]. Based on a previous report, these values indicate that the reliability and validity of the questionnaire are good and that the questionnaire is eligible for psychological evaluations [20]. There were four job burnout sublevels: zero burnout (emotional exhaustion < 25, depersonalization < 11, and reduced sense of achievement < 16), mild burnout (emotional exhaustion ≥ 25, depersonalization < 11, and reduced sense of achievement < 16; emotional exhaustion < 25, depersonalization ≥ 11, and reduced sense of achievement < 16; or emotional exhaustion < 25, depersonalization < 11, and reduced sense of achievement ≥ 16), moderate burnout (emotional exhaustion ≥ 25, depersonalization ≥ 11, and reduced sense of achievement < 16; emotional exhaustion ≥ 25, depersonalization < 11, and reduced sense of achievement ≥ 16; or emotional exhaustion < 25, depersonalization ≥ 11, and reduced sense of achievement ≥ 16), and severe burnout (emotional exhaustion ≥ 25, depersonalization ≥ 11, and reduced sense of achievement ≥ 16). The critical values of the three levels of the job burnout scale were defined as ≥25, ≥11, and ≥16.

### 2.3. MSDs

The MSD questionnaire jointly developed by Kapitan et al. was adopted in this study [21, 22]. The questionnaire, which has good reliability and validity, includes general conditions (17 items), health conditions (three sections evaluating work-related pain or discomfort in nine areas of the body (neck, shoulder, back, elbow, waist, wrist, hip, knee, and, ankle, and foot) in the past year and in the past week), and working conditions. The working conditions included movements requiring great effort (such as lifting, pushing, pulling, unfavorable posture, and trunk bending), dynamic loads (trunk movements and neck, shoulder, and wrist movements, etc.), static loads (including slight bending and severe bending), repetitive loads (i.e., work with the same movement), ergonomic environment (working environment, lack of support, slipping and falling, forward movement with tools, etc.), and environmental temperature. Most questions required a simple “yes” or “no” answer.

### 2.4. Quality Control

Quality control was monitored throughout the on-site investigations and study, including at the design stage (questionnaire design, investigators, on-site investigation, and data collation).

### 2.5. Statistical Analysis

All data were entered into the EpiData 3.0 database and analyzed using the SPSS 20.0 software package. All measurement data were reported as the mean ± standard deviation (SD). Two independent-sample *t*-tests were used to evaluate differences between two groups, whereas differences among several groups were evaluated by one-way analysis of variance (ANOVA). If an overall difference was found, Student–Newman–Keuls- (SNK-) *q* tests were used for multiple comparisons. Comparison of rates was performed using chi-squared tests. A multivariate logistic regression model of MSDs of coal mine workers was established by incorporating variables with *P* < 0.1 in univariate analysis into the logistic regression equation. Furthermore, the analysis of factors affecting job burnout and MSDs was conducted through univariate and multivariate logistic regression models. The test level was *α* = 0.05.

## 3. Results

### 3.1. Baseline Data

The baseline data for the participants are shown in Table 1. A total of 1,500 questionnaires were distributed and 1,325 valid questionnaires were returned, with a recovery rate of 88.3%. Specifically, the respondents comprised 1,220 men and 105 women, with a mean age of 42.2 ± 8.7 years and a mean length of service of 17.5 ± 10.6 years. Regarding education background, 43.8% of participants had received a junior high school education or lower, 31.5% had received a senior high school or technical secondary school education, 18.4% had received a junior college education, and 6.2% had a bachelor's degree or higher.

### 3.2. Investigation on Job Burnout among Coal Miners

In terms of job burnout among the coal mine workers, we recorded a mean score of 50.77 ± 11.93, with job burnout detected in 90.0% of participants. The emotional exhaustion score was 16.83 ± 7.77, the depersonalization score was 12.61 ± 6.90, and the reduced sense of achievement score was 21.35 ± 7.66. In terms of the degree of job burnout among the coal mine workers, 527 (39.8%) were classified as slight burnout, 581 (43.8%) were classified as moderate burnout, and 85 (6.4%) were classified as severe burnout.

### 3.3. Weekly Prevalence of MSDs among Coal Miners

The weekly prevalence of MSDs among coal mine workers is shown in Table 2. The highest weekly prevalence of MSDs among the coal mine workers was related to the waist (41.3%), followed by the neck (32.2%), shoulder (27.4%), elbow (14.4%), and ankle (14.4%). The highest rate of MSDs resulting in a request for leave of absence was related to the waist (14.8%), while the lowest rate was related to the wrist (9.4%).

### 3.4. Annual Prevalence of MSDs among Coal Mine Workers

The annual prevalence of MSDs among coal mine workers is shown in Table 3. The annual prevalence of MSDs among coal mine workers was 65.6%, which was dominated by MSDs related to the waist, neck, and shoulder and knee (juxtaposed). The highest annual prevalence of MSDs was related to the waist (50.7%), while the lowest was related to the elbow (18.8%). The highest rate of MSDs causing requests for leave of absence was related to the waist (25.7%), while the lowest rate was related to the wrist (13.4%).

### 3.5. Univariate Logistic Regression Model of MSDs of Coal Mine Workers

Details of the factors influencing MSD prevalence are shown in Table 4. The significant influencing factors identified for MSDs among coal mine workers were as follows: years of service, significant bending, forward neck tilting, maximum force operation over a period of time, repeated movements of the upper arms or fingers, slip or fall incidents, work under conditions of cold or temperature variations, and job burnout levels. The prevalence of MSDs among coal mine workers increased with the years of services. In terms of the shift system, the lowest incidence of MSDs was associated with the two-shift with three-group pattern. Job burnout was identified as a risk factor for MSDs, with severe job burnout associated with a significantly higher risk of illness compared with that of the no burnout group (*P* < 0.05).

### 3.6. Multivariate Logistic Regression Model of MSDs of Coal Mine Workers

The results of the multivariate logistic regression analysis of MSDs among coal mine workers are shown in Table 5. Significant bending, forward neck tilting, maximum force operation within a short period of time, repeated movements of the upper arms or fingers, slip or fall incidents, and work under conditions of cold or temperature variations had positive contributions to the prevalence of MSDs among coal mine workers (*P* < 0.05). Burnout increases the risk of musculoskeletal injuries. By comparing the coal miners without burnout, mild burnout, moderate burnout, and severe burnout, we found that with the increase of occupational burnout, the prevalence of musculoskeletal diseases of coal mine workers also increased. These results indicate that the higher the degree of job burnout, the greater the risk of illness.

## 4. Discussion

Coal mine workers represent a huge population in China's workforce (nearly 100,000 are employed in Xinjiang), requiring special attention regarding their physical and mental health. Many risk factors for MSDs are associated with the daily activities of coal mine workers, including the physical stress caused by dynamic and static loads, as well as ergonomic and environmental factors. The prevalence of MSDs in the general working population ranges from 20% to 40% [23–26]. Our survey showed that the annual prevalence of MSDs among coal mine workers on site is 65.6%, which is higher than that of the general working population and lower than that (78.4%) reported in 2011 by Xu [27]. As previously reported [28, 29], the prevalence of MSDs for gas station employees is 51.2% and that for nursing staff is 78.58%. The top three positions in terms of the prevalence of MSDs for nursing staff [29], automobile manufactory workers [30], oil extraction workers [31], and power plant workers [32] are the waist, neck, and shoulder, which is consistent with the results obtained in this study. The waist is an important region for normal movement of the human body. The five postures of standing, lifting, squatting, sitting, and carrying all out strain on the waist, which are inevitable aspects of all types of work, thus having a greater impact on the work once the waist has been injured. Therefore, the waist is ranked first among the positions leading to absence due to illness, followed by the ankle, possibly due the pressure imposed on this joint by the long distances traveled by coal mine workers from their homes to the mine and the requirement to remain standing for long periods of time during their working hours.

In terms of demographic factors, a previous study revealed a higher prevalence of MSDs related to the waist and neck in women than in men [31]. The prevalence of MSDs in the hip and knee also increased with the age of the participants, with the highest prevalence rate in the waist in individuals aged between 40 and 50 years. In contrast, there were no significant differences in the prevalence of MSDs at other positions. Our results showed that the prevalence of MSDs increased with the number of years of services, which is consistent with previous studies [33–35] especially after the 10 or more years of services. This phenomenon results in a more rapid increase in the prevalence of MSDs in this group than that of workers with less than 10 years of service, in contrast to slowed increase in the prevalence of MSDs when the years of service exceed 20. In general, coal mine workers, who are mainly engaged in manual labor, are prone to sickness caused by overwork under the influence of various adverse factors. However, with the experience that comes with increasing years of services, these workers learn strategies to avoid adverse working postures. On the other hand, under the influence of their own MSDs, the working efficiency of these individuals decreases and they cannot maintain their previous workload. As a result, the prevalence of MSDs increases less in the groups with 10 to 20 years of service and those with more than 20 years of service. The univariate logistic regression analysis of the groups classified according to years of services revealed that the risk of MSDs in the groups with more than 20 years and between 10 and 20 years of service was higher than that of the group with less than 10 years of services, respectively. These results confirm that the increase in the number of years of services is a risk factor for MSDs in coal mine workers. Furthermore, the results also show that the prevalence of MSDs among coal mine workers is the lowest in those employed under the system of two shifts with three groups. This employment practice was also shown to be protective against MSDs compared with that of individuals working day shifts only. The comprehensive evaluation based on the establishment of a logistic regression model revealed the existence of numerous influencing factors for MSDs among coal mine workers including significant bending, forward neck tilting, maximum force operation within a short period of time, and repeated movements of upper arms or fingers, all of which are inevitable and occur frequently in the labor process [36]. In addition, low temperature and humidity in the coal mine also have substantial influences on the work, as causes of slip and fall incidents and showing an association with environmental changes [7].

The prevalence of MSDs in various positions of coal miners with severe burnout is much higher than that of coal miners affected by job burnout to a lesser degree, suggesting that there is a certain relationship between job burnout and MSDs in this population and confirming that job burnout is a risk factor for MSDs. Psychosocial factors have been reported to have a noticeable effect on MSDs [37, 38], with adverse psychosocial factors shown to change human behavior and lead to the occurrence of MSDs [39] via a two-way feedback between psychosocial factors and MSDs [40]. Studies outside China have also shown that psychological factors and physical load simultaneously influence the prevalence of MSDs [41].

This study focused on the coal mine workers on site in Xinjiang; therefore, further studies are required to determine and verify our results in other regions. In addition, we investigated the prevalence of MSDs without clarification of the mechanism and etiology of MSDs in coal miners; thus, a cohort study is required to explore the causes and mechanisms of MSDs among coal miners.

## 5. Conclusions

In this cross-sectional study, we showed a high prevalence of MSDs among coal mine workers on site in Xinjiang. The prevalence of MSDs of the neck and waist was higher in women than in men. Furthermore, the prevalence of MSDs at each position increased with the years of service. In addition, the prevalence of MSDs in the neck of coal mine workers varied with educational background, while the prevalence in the wrist and knee varied with shift systems. Numerous factors influence the prevalence of MSDs among coal mine workers including shift system, years of services, significant bending, forward neck tilting, maximum force operation within a short period of time, repeated movements of upper arms or fingers, slip and fall incidents, work under conditions of cold or temperature variations, and job burnout, suggesting interventions to control these factors will be beneficial to both the body and mind of coal mine workers.

## Figures and Tables

**Table 1:** Baseline data.

Variables		*n*	%
Gender	Male	1,220	92.1
	Female	105	7.9
Age (years)	<30	171	12.9
	30+	254	19.2
	40+	609	46
	50+	291	22
Years of service	<10	402	30.3
	10+	305	23
	20+	618	46.6
Education	Junior high school or lower	581	43.8
	Senior high school or technical secondary	418	31.5
	Junior college	244	18.4
	Bachelor's degree or higher	82	6.2
Type of work	Coal miner	231	17.4
	Coal caver	26	2
	Coal drivage worker	238	18
	Anchor worker	11	0.8
	Craft worker	26	2
	Inspection worker	31	2.3
	Ventilation worker	48	3.6
	Gangue collector	21	1.6
	Electric locomotive driver	26	2
	Bell man	38	2.9
	Electrical fitter	158	11.9
	Plugman	18	1.4
	Operator	42	3.2
	Blaster	20	1.5
	Gas inspector	27	2
	Backman	49	3.7
	Monitor observer	22	1.7
	Loading workman	25	1.9
	Others	266	20.1
Shift system	Day shift only	490	37
	Two shifts	187	14.1
	Two shifts with three groups	536	40.5
	Three shifts with four groups	112	8.5
Marital status	Unmarried	126	9.5
	Married	1,156	87.2
	Divorced or widowed	43	3.2

**Table 2:** Weekly prevalence of MSDs among coal mine workers.

Localizations	Position	MSD		Leave of absence due to MSD	
		Number of patients	Weekly prevalence	Requests for leave of absence	Absence rate due to MSD
Upper limb	Neck	427	32.20%	43	10.10%
	Shoulder	363	27.40%	37	10.20%
	Back	300	22.60%	33	11.00%
	Elbow	191	14.40%	25	13.10%
	Wrist	245	18.50%	23	9.40%
Lower limb	Waist	547	41.30%	81	14.80%
	Hip	146	11.00%	19	13.00%
	Knee	328	24.80%	39	11.90%
	Ankle	191	14.40%	28	14.70%

**Table 3:** Annual prevalence of MSDs among coal mine workers.

Localizations	Position	Illness		Request for leave of absence due to illness	
		Number of patients	Annual prevalence	Requests for leave of absence	Absence rate due to illness
Upper limb	Neck	528	39.80%	85	16.10%
	Shoulder	448	33.80%	57	12.70%
	Back	359	27.10%	59	16.40%
	Elbow	249	18.80%	42	16.90%
	Wrist	314	23.70%	42	13.40%
Lower limb	Waist	672	50.70%	173	25.70%
	Hip	199	15.00%	39	19.60%
	Knee	448	33.80%	86	19.20%
	Ankle	289	21.80%	61	21.10%

**Table 4:** Factors influencing prevalence of MSD last year.

Variables		Prevalence of MSD last year	*P*	OR	95% CI	
					Upper limit	Lower limit
Gender			0.273			
	Male	65.20%		1		
	Female	70.00%		1.276	0.826	1.973
Age (years)			0.097			
	<30	59.60%		1		
	30+	63.00%		1.151	0.774	1.714
	40+	68.80%		1.492	1.051	2.118
	50+	64.60%		1.235	0.837	1.821
Years of service			0.001			
	<10	58.50%		1		
	10+	67.20%		1.457^∗^	1.068	1.988^∗^
	20+	69.40%		1.613^∗∗^	1.241	2.096
Education			0.689			
	Junior high school or lower	63.90%		1		
	Senior high school or technical secondary	67.00%		1.148	0.881	1.497
	Junior college	66.40%		1.118	0.816	1.532
	Bachelor's degree or higher	68.30%		1.219	0.743	2.000
Shift system			0.002			
	Day shift only	67.30%		1		
	Two shifts	71.70%		1.226	0.847	1.774
	Two shifts with three groups	60.10%		0.730^∗^	0.565	0.942
	Three shifts with four groups	74.10%		1.338	0.873	2.205
Significant bending			<0.001			
	No	54.10%		1		
	Yes	75.10%		2.723^∗∗^	2.141	3.462
Forward neck tilting			<0.001			
	No	53.10%		1		
	Yes	76.80%		2.967^∗∗^	2.337	3.767
Maximum force operation within a short period of time			<0.001			
	No	55.00%		1		
	Yes	75.30%		2.655^∗∗^	2.088	3.376
Repeated movement of upper arms or fingers			<0.001			
	No	56.80%		1		
	Yes	71.90%		1.941^∗∗^	1.538	2.450
Slip or fall incidents			<0.001			
	No	55.00%		1		
	Yes	76.20%		2.744	2.155	3.495
Work under conditions of cold or temperature variations			<0.001			
	No	52.00%		1		
	Yes	74.40%		2.688^∗∗^	2.121	3.405
Burnout level			0.001			
	Zero burnout	67.40%		1		
	Mild burnout	66.20%		0.904	0.599	1.365
	Moderate burnout	61.80%		0.743	0.495	1.117
	Severe burnout	84.70%		2.530	1.225	5.099

Note: ^∗^*P* < 0.05; ^∗∗^*P* < 0.01.

**Table 5:** Multivariate logistic regression analysis of prevalence of MSD last year of coal mine workers.

Variables	Variable assignment	*B*	Wald	*P*	OR	95% CI	
						Upper limit	Lower limit
Shift system	Day shift only		25.635	<0.001	1		
	Two shifts	0.092	0.427	0.514	1.096	0.832	1.445
	Two shifts with three groups	-0.437	3.294	0.070	0.646	0.403	1.306
	Three shifts with four groups	0.811	17.294	<0.001	2.271	1.543	3.342
Years of services	<10		31.662	0.01	1		
	10+	1.306	33.332	0.000	3.690	2.369	5.748
	20+	1.101	8.251	0.004	3.008	1.419	6.377
Significant bending		0.724	13.395	<0.001	2.062	1.400	3.038
Forward neck tilting		0.565	5.657	0.017	1.572	1.071	2.281
Maximum force operation within a short period of time		0.543	7.398	0.007	1.7222	1.164	2.547
Repeated movement of upper arms or fingers		0.402	4.575	0.032	1.495	1.034	2.161
Slip or fall incidents		0.117	8.475	0.004	1.124	1.039	1.216
Work under conditions of cold or temperature variations		0.647	12.915	<0.001	1.911	1.342	2.720
Job burnout	Zero burnout		11.462	0.009	1		
	Mild burnout	0.400	4.159	0.041	1.492	1.016	2.191
	Moderate burnout	0.617	10.317	0.001	1.852	1.267	2.708
	Severe burnout	0.694	5.935	0.015	2.001	1.145	3.496

## Data Availability

The data used to support the findings of this study are available from the corresponding author upon request.
